# High-throughput genome sequencing of two *Listeria monocytogenes *clinical isolates during a large foodborne outbreak

**DOI:** 10.1186/1471-2164-11-120

**Published:** 2010-02-18

**Authors:** Matthew W Gilmour, Morag Graham, Gary Van Domselaar, Shaun Tyler, Heather Kent, Keri M Trout-Yakel, Oscar Larios, Vanessa Allen, Barbara Lee, Celine Nadon

**Affiliations:** 1 National Microbiology Laboratory, Public Health Agency of Canada, Winnipeg, MB, Canada; 2 Department of Medical Microbiology and Infectious Diseases, University of Manitoba, Winnipeg, MB, Canada; 3 Public Health Laboratories, Ontario Agency for Health Protection and Promotion, Toronto, ON, Canada; 4 Canadian Food Inspection Agency, Ottawa, ON, Canada

## Abstract

**Background:**

A large, multi-province outbreak of listeriosis associated with ready-to-eat meat products contaminated with *Listeria monocytogenes *serotype 1/2a occurred in Canada in 2008. Subtyping of outbreak-associated isolates using pulsed-field gel electrophoresis (PFGE) revealed two similar but distinct *Asc*I PFGE patterns. High-throughput pyrosequencing of two *L. monocytogenes *isolates was used to rapidly provide the genome sequence of the primary outbreak strain and to investigate the extent of genetic diversity associated with a change of a single restriction enzyme fragment during PFGE.

**Results:**

The chromosomes were collinear, but differences included 28 single nucleotide polymorphisms (SNPs) and three indels, including a 33 kbp prophage that accounted for the observed difference in *Asc*I PFGE patterns. The distribution of these traits was assessed within further clinical, environmental and food isolates associated with the outbreak, and this comparison indicated that three distinct, but highly related strains may have been involved in this nationwide outbreak. Notably, these two isolates were found to harbor a 50 kbp putative mobile genomic island encoding translocation and efflux functions that has not been observed in other *Listeria *genomes.

**Conclusions:**

High-throughput genome sequencing provided a more detailed real-time assessment of genetic traits characteristic of the outbreak strains than could be achieved with routine subtyping methods. This study confirms that the latest generation of DNA sequencing technologies can be applied during high priority public health events, and laboratories need to prepare for this inevitability and assess how to properly analyze and interpret whole genome sequences in the context of molecular epidemiology.

## Background

*Listeria monocytogenes *is a Gram-positive, facultative intracellular bacterial pathogen that can cause severe disease in humans, other mammals and birds [1]. Human listeriosis is relatively rare despite our likely frequent encounters with *L. monocytogenes*, which is ubiquitously present in the environment (including water, soil, vegetation), farm and rural environments, and urban environments [2-6]. The vast majority of human listeriosis is foodborne and the most commonly implicated vehicles are ready-to-eat food products such as meat, dairy, seafood, and fresh produce that are contaminated with *L. monocytogenes *during processing [6,7]. *L. monocytogenes *can be introduced into food processing facilities and food products due to cross-contamination with environmental sources or from the feces of food production animals. *L. monocytogenes *can persist within food processing environments for long periods of time, due in part to its ability to grow at wide-ranging temperatures and pH (0.4°C to 45°C, pH 4 to 9.6) and the ability to form biofilms promoting adherence to food processing surfaces [8-11]. The persistence of a single subtype of *L. monocytogenes *in processing facilities or on equipment has been reported from several months to more than 10 years [12,13].

Given the widespread occurrence of *L. monocytogenes*, subtyping of clinical and food isolates is required to establish epidemiologic links during routine surveillance, outbreak investigations, and for source tracking. There are 13 known serotypes of *L. monocytogenes *but the vast majority of human disease cases are caused by strains belonging to serotypes 4b, 1/2a, and 1/2b, severely limiting the utility of this subtyping method for differentiating *L. monocytogenes *[14]. Large clonal outbreaks owing to contaminated food sources such as coleslaw, milk, cheese, hot dogs and deli meats, have been predominately caused by serotypes 4b and 1/2a strains [15]. Accordingly, additional subtyping methods are required to better characterize outbreak isolates. Several molecular subtyping methods have been developed and applied to *L. monocytogenes*, including pulsed-field gel electrophoresis (PFGE), ribotyping, multilocus variable-number tandem repeat analysis (MLVA), and sequence-based subtyping [14,16,17]. PFGE has been adopted by PulseNet as the internationally standardized method for molecular subtyping of *L*.*monocytogenes *and has been essential in the detection and investigation of listeriosis outbreaks in Canada and worldwide [18-20].

DNA sequencing has enabled analyses of *L. monocytogenes *genomes and furthered the understanding of this pathogen's biology and phylogeny. Comparative analyses of genome sequences have elucidated the genetic differences between *L. monocytogenes *serotypes, the acquisition and evolution of virulence and pathogenic traits among *Listeria *spp., and the genetic basis underlying the unique survival and growth characteristics of *L. monocytogenes *[21-23]. Genome sequencing has also permitted the development of multi-locus sequence typing (MLST) protocols, which in combination with other typing methods, have validated three evolutionary lineages for *L. monocytogenes *[24-29]. The lineages reflect the serotype distribution, with serotypes 1/2b, 3b and 4b segregating to lineage I and serotypes 1/2a and 1/2c segregating to lineage II. Risk assessments based upon genetic lineages have suggested that *L. monocytogenes *subtypes may have unique ecological niches and may also differ in their pathogenic potential or host adaptations [30,31].

The majority of available bacterial genome sequences have been generated using the Sanger chain termination sequencing chemistries. Although this technology was instrumental in the emergence of the field of genomics, it is time and resource intensive [32,33]. Several post-Sanger sequencing technologies (also referred to as next-generation sequencing technologies) have since been developed that enable extremely rapid whole-genome sequencing and a broader application of comparative genomics [33,34]. The high-throughput pyrosequencing method commercialized as the 454 or GS FLX™ platform has been applied for rapidly determining the genome sequences of bacterial isolates.

*L. monocytogenes *serotype 1/2a caused a nationwide outbreak of listeriosis associated with ready-to-eat meat products in Canada during the summer of 2008. The outbreak resulted in 22 deaths and at least 57 illnesses. Whereas the majority of outbreak-associated isolates (human clinical and food isolates) had an indistinguishable *Asc*I PFGE pattern, one clinical isolate from Ontario and several isolates from the affected food production facility exhibited an *Asc*I PFGE pattern that differed by a single restriction enzyme fragment compared to the primary outbreak strain. We report herein the first real-time application of whole-genome sequencing during an active listeriosis outbreak investigation. High-throughput pyrosequencing was applied to characterize two outbreak-associated isolates of *L. monocytogenes *in order to obtain a thorough genetic characterization of the outbreak strains and to determine the genetic basis for the single *Asc*I restriction fragment change.

## Results and Discussion

### Primary outbreak isolate harbors unique plasmid pLM5578 and prophage ϕLMC1

Public health responses to foodborne outbreaks require bacterial subtyping data to definitively link affected individuals with contaminated food sources and other suspected patients. The genetic relatedness between clinical and food isolates is routinely determined using methods such as PFGE, however, with the latest generation of DNA sequencing technologies it is theoretically possible to now determine the complete genetic blueprint of bacterial isolates within the scope of an outbreak. In this study, draft genome sequences suitable for comparative analysis between outbreak isolates and to reference genomes were available within three days. These data allowed for discovery of novel prophage and genomic islands, and the subsequent sequence closure and the confirmation of sequence variants allowed for annotation of polymorphisms and other traits associated with micro-diversity.

**Figure 1:**
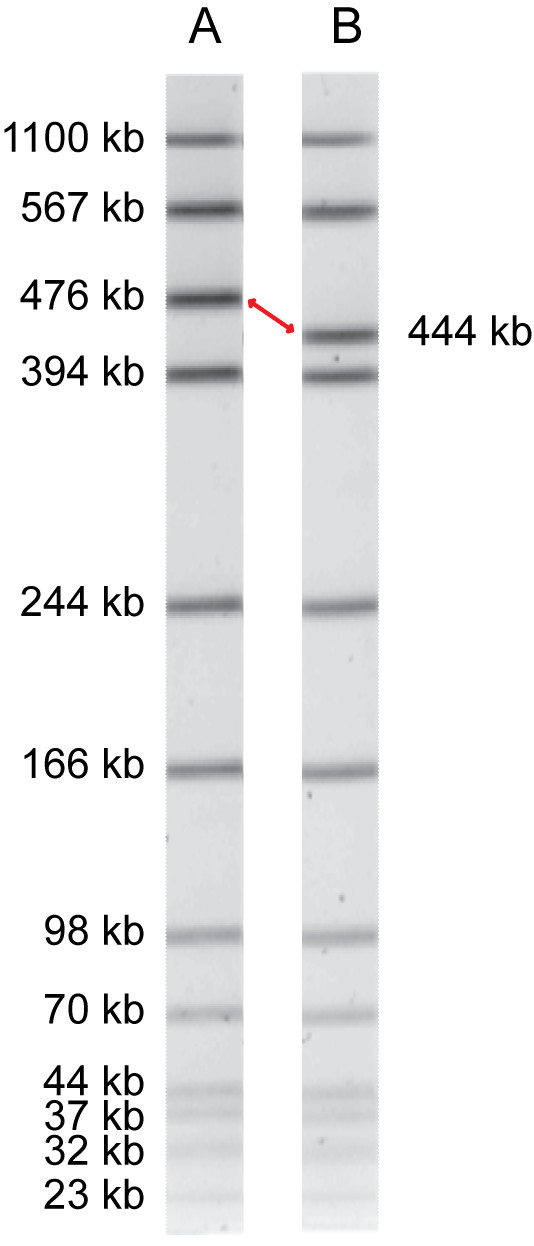
***Asc*I pulsed-field gel electrophoresis of two *Listeria monocytogenes *PFGE patterns associated with a large foodborne outbreak**. A: LMACI.0040; B: LMACI.0001. Restriction fragment sizes were estimated using BioNumerics relative to the control standard. Unmatched bands are highlighted (red arrow).

**Figure 2:**
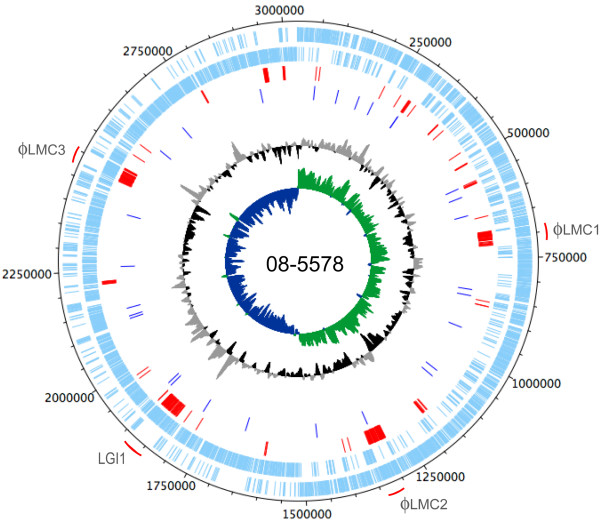
**Circular map and genetic features of *Listeria monocytogenes *isolate 08-5578**. The outer ring denotes genetic coordinates, and prophage and the novel 50 kbp *Listeria *genomic island (LGI1) are indicated in grey text. Prophage ϕLMC1 is not encoded within isolate 08-5923. Light blue bars (2^nd ^and 3^rd ^rings) denote coding sequences on the positive and negative strands, respectively. Red bars (4^th ^ring) denote those coding sequences present in 08-5578 but absent in the genome sequence of strain EGDe. Dark blue bars (5^th ^ring) indicate confirmed single nucleotide polymorphisms between isolate 08-5578 and 08-5923. The black/grey and blue/green plots indicate G+C content and G+C skew, respectively.

**Figure 3:**
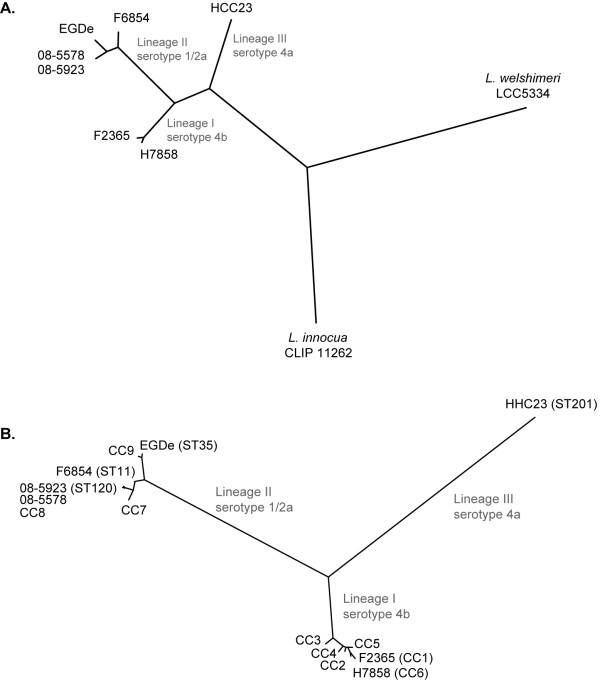
**Maximum likelihood phylogenetic trees determined for *Listeria *genome sequences (A) and MLST loci (B)**. *L. monocytogenes *lineages and serotypes are indicated (grey text). 'CC' denotes clonal complexes and 'ST' denotes sequence types. Strain F2365 was isolated from a 1985 California cheese outbreak; H7858 from a 1998-9 Multistate hotdog oubreak; F6854 from a 1988 Oklahoma turkey hot dog sporadic case; EGDe is a laboratory strain passaged from an animal isolate from 1924.

Clinical isolate 08-5578 was selected as the reference outbreak strain (serotype 1/2a; PFGE patterns LMACI.0040 and LMAAI.0001; Table 1) and clinical isolate 08-5923 differed only in the *Asc*I restriction pattern (LMACI.0001; Fig. 1). High-throughput pyrosequencing was performed on both isolates and closed circular chromosomal sequences were obtained for each isolate (Table 2; Fig. 2). A 77 kbp contig present in the 08-5578 sequence assembly but absent in 08-5923 was identified as a circular plasmid and designated pLM5578 (Table 2). Comparison of the chromosomal sequences for isolates 08-5578 and 08-5923 with publicly available *Listeria spp. *genomes (Fig. 3A) and with select loci used for MLST (Fig. 3B) indicated that these isolates were members of evolutionary lineage II and clonal complex 8 (discussed further below).

**Table 1:** Bacterial isolates used in this study and results of PCR-based screening assays.

				Pulsed-field gel electrophoresis		Prophage ϕLMC3^b^		pLM5578				
							
Isolate No.	Source	Specimen type^a^	Serotype	*Asc*I	*Apa*I	terminase	tail protein	*virD4*	*fic*	*buk*^c^	*gltX*^d^	SNPs^e^
08-5578	Human	Blood	1/2a	LMACI.0040	LMAAI.0001	+	+	+	+	FS	- 21 bp	1
08-5923	Human	Blood	1/2a	LMACI.0001	LMAAI.0001	-	-	-	-	WT	WT	27
08-6040	Food	RTE meat	1/2a	LMACI.0040	LMAAI.0001	+	+	+	+	FS	- 21 bp	1
08-6055	Food	RTE meat	1/2a	LMACI.0040	LMAAI.0001	+	+	-	-	FS	- 21 bp	1
08-6135	Human	CSF	1/2a	LMACI.0040	LMAAI.0001	+	+	+	+	FS	- 21 bp	1
08-6567	Environment	Food processing	1/2a	LMACI.0040	LMAAI.0001	+	+	+	+	FS	- 21 bp	1
08-6061	Food	RTE meat	1/2a	LMACI.0040	LMAAI.0001	+	+	+	+	FS	- 21 bp	1
08-6421	Human	Blood	1/2a	LMACI.0040	LMAAI.0001	+	+	+	+	FS	- 21 bp	1
08-5828	Human	Blood	1/2a	LMACI.0040	LMAAI.0001	+	+	-	-	FS	- 21 bp	1
08-7374	Environment	Food processing	1/2a	LMACI.0001	LMAAI.0001	-	-	-	-	FS	WT	0
08-7376	Environment	Food processing	1/2a	LMACI.0001	LMAAI.0001	-	-	-	-	FS	WT	0
08-7381	Environment	Food processing	1/2a	LMACI.0001	LMAAI.0001	-	-	-	-	FS	WT	0
08-7382	Environment	Food processing	1/2a	LMACI.0001	LMAAI.0001	-	-	-	-	FS	WT	0

a. 'RTE', ready to eat; 'CSF', cerebrospinal fluid

b. '+', amplicon of expected size detected using PCR using oligonucleotides described in Additional file 6; '-', no amplicon detected

c. 'FS', frameshift resulting in a truncated coding sequence encoding butyrate kinase; 'WT', wild type. Confirmed by Sanger-based DNA sequencing of amplicons generated by high fidelity PCR.

d. 'WT', wild type; '-21 bp', in-frame deletion of 7 codons. Confirmed by Sanger-based DNA sequencing of amplicons generated by PCR.

e. Number of SNP's relative to the hypothetical last common ancestor presented in Fig. 6. Sanger-based DNA sequence confirmation of SNPs was completed after PCR amplification using oligonucleotides described in Additional file 6.

**Table 2:** General genomic characteristics of *Listeria monocytogenes *isolates 08-5578, 08-5923, and plasmid pLM5578.

	08-5578	pLM5578	08-5923
Genome size (bp)	3 032 288	77 054	2 999 054
G/C content	37.96	36.59	37.96
No. of predicted coding sequences	3010	79	2966
Average length of coding sequences (bp)	900	833	903
Coding percentage	89.3	85.4	89.3
rRNA loci	13	0	13
Bacteriophage	3	0	2

**Figure 4:**
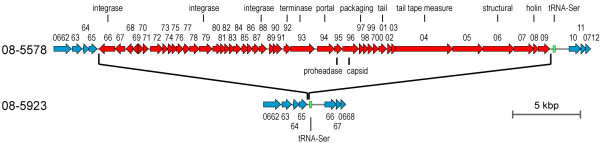
**Schematic of a 33 kbp prophage unique to *Listeria monocytogenes *isolate 08-5578**. Blue-colored loci represent a contiguous segment within the isolate 08-5923 genome. Similarly, regions flanking the prophage of isolate 08-5578 are also denoted in blue. The interrupting contiguous coding sequences (red) represent prophage ϕLMC1 in 08-5578. The tRNA-Ser gene is denoted with a green box. Putative phage-related functions or structures are indicated above the locus tag identifiers. A nucleotide scale bar for size estimation is included.

The *Asc*I restriction patterns of 08-5578 and 08-5923 were indistinguishable, except for a single band shift of approximately 32 kbp (from 476 kbp in 08-5578 to 444 kbp in 08-5923; Fig. 1). Accordingly, comparative sequence alignment revealed a 33 kbp contiguous region integrated adjacent to (but not disrupting) the tRNA-Ser gene that was unique to 08-5578 and encoding several putative bacteriophage-related coding sequences (designated ϕLMC1) (Fig. 4). BLAST analyses indicated that this prophage was unique among sequenced *Listeria *genomes, although it was comprised of coding sequences similar to determinants from several known phages associated with Gram-positive microbes, including *L. monocytogenes *phages A006, A118 and B025 (see Additional file 1). The presence of prophage ϕLMC1 accounted for the *Asc*I restriction pattern difference between isolates 08-5578 and 08-5923.

**Figure 5:**
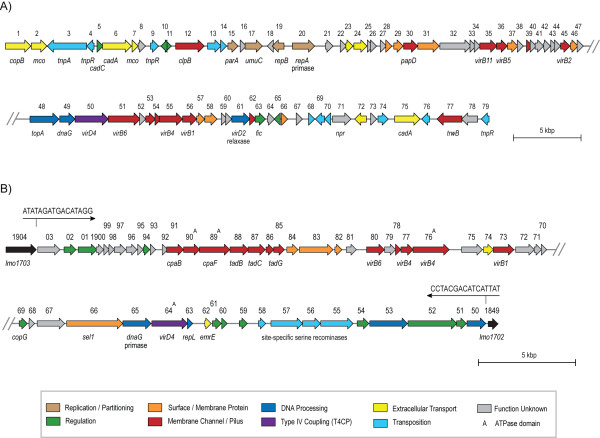
**Genetic organization and predicted functions of pLM5578 (A) and the *Listeria *genomic island 1 (LGI1) (B)**. Both sequences represent contiguous genetic regions but are split onto two lines for visual clarity, with the site of the artificial segmentation denoted with angled lines. Locus tags are as denoted above the CDS map and predicted gene names (italics) denoted below. Coding sequences are color-coded based on predicted function, with the legend included in the inset. Black-colored coding sequences are similar to *L. monocytogenes *EGDe (locus tags, *lmo*) and the imperfect inverted 16 bp repeats surrounding the genomic island are indicated. A nucleotide scale bar for size estimation is included.

The extrachromosomal circular plasmid (pLM5578) harbored in isolate 08-5578 encoded putative determinants for replication, partitioning, heavy metal resistance and DNA translocation (Fig. 5a, and Additional file 2). Many of these features were homologous to sequences encoded by plasmids from *L. innocua *(pLI100), *L. monocytogenes *serotype 4b isolate H7858 (pLM80) and *Bacillus anthracis *(pXO2), including a replication and regulation control center (pLM5578_17 - 20). Two regions of pLM5578 were related to heavy metal resistance, including a transposon-associated *cadA *that exhibited high sequence identity to pLI100. A second, distinct region unique to pLM5578 was comprised of *cadA *(second copy) and *cadC *genes, with the latter encoding a cadmium-efflux ArsR-family regulatory accessory protein (repressor) homolog. Resistance to the heavy metal cadmium has been recently discovered to be associated with resistance to quaternary ammonium compounds (sanitizers), which may contribute to *L. monocytogenes *persistence in food production facilities [35]. Notably, several coding sequences exhibited sequence similarity to type IV secretion systems (T4SS), including DNA processing (*virD2*), DNA coupling (*virD4*) and membrane channel formation (*virB1, -B4, -B5, -B6, -B11*) (see Additional file 2). Additional predicted membrane-associated proteins were present within this region, and all coding sequences were oriented in the same direction (Fig. 5A), which is consistent with the operon-style organization of most known T4SS. Varying degrees of similarity between pLI100, pLM80 and pXO2 plasmids have already been established [21], and our data further support a chimeric nature for *Listeria *plasmids.

### pLM5578 and ϕLMC1 exhibit non-homogeneous distribution within a wider panel of outbreak-associated isolates

During the outbreak, serotype 1/2a *L. monocytogenes *isolates were recovered from clinical samples, contaminated food products and the food processing environment that shared the two *Asc*I PFGE patterns observed for the sequenced isolates (Table 1). To determine the distribution of ϕLMC1 and pLM5578 amongst outbreak-associated isolates, PCR-based screening was conducted by targeting ϕLMC1 loci encoding a putative phage terminase large subunit and phage tail tape measure protein, and pLM5578 loci encoding *virD4 *and *fic *(Table 1). All isolates with *Asc*I pattern LMACI.0001 lacked the ϕLMC1 determinants by PCR, confirming that this novel sequence insertion accounts for the restriction enzyme pattern shift relative to PFGE pattern LMACI.0040. Bacteriophage insertions have previously been recognized as contributing to macromolecular genetic diversity between related outbreak-associated *L. monocytogenes *strains [12,36].

The PCR screening assays for plasmid determinants confirmed that pLM5578 was absent in isolate 08-5923, and this plasmid was also absent in other outbreak-related isolates (independent of PFGE profile), including one clinical isolate (Table 1). The sequence of pLM5578 revealed an absence of *Asc*I restriction sites and presence of a single *Apa*I restriction site, but this element did not contribute to the PFGE patterns resulting from these enzymes. In contrast, plasmid pLM80 (containing a single *Apa*I recognition site) had been reported as visible in *Apa*I PFGE digests [36]. Just as a variable presence of plasmid pLM5578 was observed in this Canadian outbreak, plasmid pLM80 was sporadically present in isolates during the 1998-99 multi-state serotype 4b frankfurter USA outbreak [36]. Lack of stable vertical transmission of pLM5578 during the outbreak or laboratory culture (despite the presence of a partitioning determinant, pLM5578_15) might account for plasmid absence. Alternatively, pLM5578 may have been carried only in a subset of *L. monocytogenes *strains that subsequently caused the outbreak. Presence of determinants related to bacterial conjugation (Fig. 5A) further confounds any speculation on the transmission of this plasmid.

### Whole genome comparisons enable construction of an evolutionary model of the outbreak isolates

The genomes of 08-5578 and 08-5923 were collinear, and with the exception of the prophage and plasmid unique to 08-5578, all genetic diversity was accounted for by 2 short indels and 28 confirmed SNPs. The two indels included a single C/T base pair insertion in 08-5578 that introduced a frameshift truncation into coding sequence LM5578_1509 (*buk*, encoding a putative butyrate kinase). Secondly, there was a 21 bp (7 codon) in-frame deletion in the *gltX *gene (LM5578_0279) of 08-5578 encoding a putative glutamyl-tRNA synthetase. DNA sequencing confirmed that this *gltX *deletion was present in all examined LMACI.0040 isolates but that a wild type allele was present in all isolates typed as LMACI.0001 (Table 1). Alternatively, a wild type *buk *gene was only observed in isolate 08-5923 and a truncated butyrate kinase was encoded in all other isolates, independent of PFGE pattern (Table 1). Directed PCR and DNA sequencing of the SNP sites within the panel of clinical, food and environmental isolates was also performed (Table 1). Of the 28 SNPs, 27 were only present in isolate 08-5923. The singular remaining non-coding SNP was observed in each of LMACI.0040 isolates (at intergenic coordinate 2691224 of 08-5578) but was absent in all of the LMACI.0001 isolates, including 08-5923.

The 27 SNPs observed in the genome of clinical isolate 08-5923 included 20 non-synonymous, 5 synonymous and 2 intergenic mutations (Table 3). Such a predominance of non-synonymous changes suggests that isolate 08-5923 was under strong positive selection for functional divergence rather than purifying selection (wherein synonymous exceed non-synonymous mutation rates). The non-synonymous mutations occurred in nine COG categories, including four changes related to transcription, but no mutations occurred in COGs related to DNA repair, recombination or replication (Table 3). Only four non-synonymous changes represented functionally analogous amino acid changes (e.g, I/L/V/G) and none were in exported proteins carrying signal peptides. To place this finding into context, *Listeria *species have been reported as having the strongest purifying selection (elevated synonymous mutation rates) of all the prokaryotes following comparison of multiple completed genome sequences [37]. Furthermore, genome sequencing of four serotype 1/2a lineage II isolates from an endemic clone contaminating a single food production facility from 1988 to 2000 revealed only 11 total SNPs outside of the bacteriophage determinants, and a maximum of 8 SNPs were observed between any two given strains [12]. This low number of SNPs in an endemic clone spanning over 12 years suggested that non-controlled natural populations of *L. monocytogenes *are stable and exhibit limited genetic micro-diversity [12]. If a similar mutation rate occurred in the serotype 1/2a *L. monocytogenes *isolates recovered during this 2008 Canadian outbreak, passage of several decades would be expected in order to attain 28 total SNPs. We are assuming that 08-5923 shares a very recent ancestor as 08-5578 and thus speculate that 08-5923 was subjected to pressures resulting in elevated mutation rates and an abundance of non-synonymous changes.

**Table 3:** Single nucleotide polymorphisms (SNPs) identified within chromosomal sequences of isolate 08-5923 relative to 08-5578.

SNP position	CDS/Intergenic	gene	08-5578 sequence	08-5923 sequence	08-5578 codon	08-5923 codon	08-5578 residue	08-5923 residue	Predicted Product
47737	non-synonymous		T	C	ATA	ACA	I	T	hypothetical protein
113283	non-synonymous		(G)	(A)	(GAT)	(AAT)	D	N	ATP-binding cassette, subfamily B
172841	synonymous	*yaaQ*	A	G	CCA	CCG	P	P	hypothetical protein
213957	non-synonymous	*fusA*	T	A	TTC	TAC	F	Y	elongation factor G
291652	non-synonymous	*cytR*	T	G	AGT	AGG	S	R	transcriptional regulator, LacI family
291653	non-synonymous	*cytR*	G	T	GTG	TTG	V	L	transcriptional regulator, LacI family
552482	non-synonymous	*hsdR*	C	G	TGC	TGG	C	W	type I restriction enzyme, R subunit
577443	non-synonymous	*celF*	A	G	GAC	GGC	D	G	6-phospho-beta-glucosidase
630182	non-synonymous		C	A	ACA	AAA	T	K	hypothetical protein
833717	non-synonymous	*araR*	(A)	(T)	(AAC)	(TAC)	N	Y	arabinose operon transcriptional repressor
850721	intergenic		G	C					
940888	non-synonymous	*pgcA*	A	G	GAA	GGA	E	G	phosphomannomutase
1076779	synonymous	*ykuQ*	C	T	TTC	TTT	F	F	Tetrahydrodipicolinate N-succinyltransferase
1096500	non-synonymous		A	T	ATA	TTA	I	L	hypothetical protein
1319530	non-synonymous		T	G	TGT	GGT	C	G	hypothetical protein
1462121	non-synonymous	*pta2*	C	T	GCT	GTT	A	V	phosphate butyryltransferase
1660409	synonymous	*hemL*	(C)	(G)	(CGC)	(CGG)	R	R	glutamate-1-semialdehyde aminotransferase
1787404	non-synonymous		T	A	TTT	TTA	F	L	hypothetical protein
1901622	non-synonymous		(T)	(C)	(GTA)	(GCA)	V	A	sigma-54 dependent transcriptional regulator
1910870	non-synonymous		(C)	(G)	(CGC)	(GGC)	R	G	hypothetical protein
2117257	non-synonymous	*aroA*	(T)	(G)	(GTC)	(GGC)	V	G	3-phosphoshikimate 1-carboxyvinyltransferase
2124375	non-synonymous	*hepT*	(C)	(T)	(GCA)	(GTA)	A	V	trans-hexaprenyltranstransferase
2146277	non-synonymous	*punA*	(A)	(G)	(ATA)	(GTA)	I	V	purine-nucleoside phosphorylase
2261927	intergenic		C	A					
2404315	non-synonymous		G	A	AGT	AAT	S	N	hypothetical protein
2603164	synonymous		(T)	(G)	(CTT)	(CTG)	L	L	D-methionine transport system ATP-binding protein
2691224	intergenic		G	T					
2929661	synonymous	*atpA*	(A)	(G)	(TCA)	(TCG)	S	S	F-type H+-transporting ATPase alpha chain

SNP coordinates are in relation to the genome of 08-5578 and the nucleotide composition refers to the positive DNA strand unless the SNP lies within a coding sequence on the negative (complementary) strand, and in these instances are marked in brackets.

**Figure 6:**
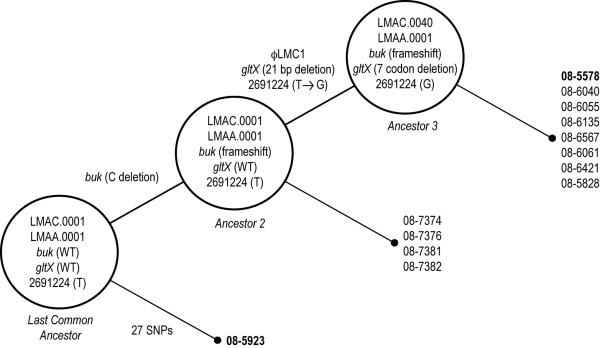
**Evolutionary model for the *Listeria monocytogenes *isolates recovered during the 2008 nation-wide foodborne outbreak**. Predicted mutational events are indicated on the diagonal lines, genotypes of the resulting lineages are denoted within circles, and isolates representative of those lineages are indicated to the right of solid dots. Sequenced isolates are denoted with bold text.

We propose a model to describe the chromosomal evolution of strains involved in this nation-wide outbreak using the distribution and segregation of genetic traits such as SNPs, indels and prophage (Fig. 6). This model assumes that the last common ancestor (LCA) of all strains encoded wild type *buk *and *gltX *loci, ϕLMC1 was absent, and the wild type state of all SNP positions was represented by the sequences in 08-5578 with the exception of intergenic SNP at 2691224. Isolate 08-5923 is predicted to be a direct descendent of the LCA, as it is the only isolate encoding a wild type *buk *gene. The remainder of the LMACI.0001 isolates differed from the LCA by only the single frameshift in *buk*, generating ancestor 2 (Fig. 6). From ancestor 2, acquisition of three independent traits (ϕLMC1, the 21 bp deletion in *gltX *and the intergenic SNP at coordinate 2691224) occurred, resulting in a third lineage defined by the LMACI.0040 PFGE pattern. In light of the high numbers of non-synonymous SNPs in 08-5923 and macromolecular genetic changes such as bacteriophage integration in the lineage represented by 08-5578, we speculate that three distinct, but highly related strains were circulating in this outbreak (Fig. 6). The diversifying SNPs in 08-5923 may have occurred in response to adverse environmental conditions prior to infection or host-specific conditions during infection, and ϕLMC1 likely arose by acquisition from other natural populations in the affected production facility.

### Genome sequence alignments reveal that the outbreak isolates belong to evolutionary lineage II - clonal complex 8

Phylogenetic comparison to *Listeria *genome sequences representing each of the three *L. monocytogenes *evolutionary lineages, *L. welshimeri *and *L. innocua *indicated that 08-5578 and 08-5923 are both lineage II strains (Fig. 3A). The most closely related genome was strain EGDe, a serotype 1/2a lab-passaged animal isolate from 1924 [22]. For detection of large-scale genomic rearrangements, a dot plot comparison of 08-5578 versus the EGDe genome sequence was performed (see Additional file 3). This revealed that the common chromosomal sequences were collinear, with the exception of a large 50 kbp inverted segment around the putative origin of replication (coordinate 1 of EGDe) (see Additional file 3). Symmetric inversions around the origin of replication have previously been observed between other bacterial genome sequences [38]. In contrast to only 28 SNPs being observed between 08-5578 and 08-5923, 24660 high quality SNPs and 941 estimated indels were revealed in a comparison of 08-5578 and EGDe (data not shown). These included 17874 synonymous SNPs, 4692 non-synonymous SNPs and 475 indels occurring within 1214 predicted coding sequences, with 1 to 167 non-synonymous SNPs and/or indels occurring per gene. Moreover, two large contiguous regions present in both 08-5578 and 08-5923 represent probable prophages (designated ϕLMC2 and ϕLMC3) that are absent in EGDe (Fig. 2, see Additional file 3). ϕLMC2 was highly related to phage B025, and ϕLMC3 was inserted at *comK *and related to *Listeria *phage A006.

Comparative analysis of the regions used for multi-locus sequence typing (MLST) indicated that isolates 08-5578 and 08-5923 are more closely related to lineage II clonal complex 8 (CC8) than to EGDe (Fig. 3B). The MLST determinants used by Ragon *et. al. *[24] were extracted *in silico *from the genome sequences and 08-5923 was identical to a previously characterized CC8, sequence type 120 (ST120) strain (encoding *abcZ *allele 5, *bglA *allele 6, *cat *allele 2, *dapE *allele 29, *dat *allele 5, *ldh *allele 3, *lhkA *allele 1). The extracted sequences for 08-5578 were also identical to ST120 except for one SNP within the *abcZ *locus not currently listed in the Institute Pasteur's *L. monocytogenes *MLST database. The sole previous member of ST120 was identified as a clinical serotype 1/2a isolate derived from a CNS infection in New Zealand in 1995 [24]. Phylogenetic trees based on MLST and whole genome sequences were relatively congruent when considering evolutionary lineages, however genome sequences provide a more precise placement relative to other examined isolates. The pyrosequencing data also provided the opportunity to analyze the entire genome for micro and macro-diversity, including the full repertoire of phylogenetically relevant loci.

### Virulence-associated determinants of the outbreak isolates informs lineage- and strain-specific virulence potential

The surface protein internalin, encoded by the *inlA *locus, is a principal virulence determinant promoting mammalian host cell invasion via interaction with E-cadherin on epithelial cells [39]. Truncation variants of *inlA *have been associated with decreased pathogenicity and significantly reduced invasiveness for human intestinal epithelial cells [40,41]. Using nomenclature assigned by Ragon *et al. *[24], isolates 08-5578 and 08-5923 both encode a full length *inlA *allele type 2 that has previously been observed in isolates related to human illness [24]. This singular determinant however cannot account for differences in pathogenicity since foodborne isolates have been observed with intact *inlA *loci [42] and *inlA *allele 2 is also encoded by environmental isolates [43]. *L. monocytogenes *does encode multiple additional internalin and internalin-like coding sequences, although the function of each paralog is currently unknown [21,44]. Isolates 08-5578 and 08-5923 both encode *inlB, inlC, inlC2, inlD, inlE, inlF, inlG, inlI, inlJ *and 11 other leucine-rich internalin-like coding sequences (see Additional file 4). The presence of an intact *inlA *locus and this compilation of other internalin-like loci may account in part for the pathogenicity of the serotype 1/2a outbreak strains sequenced in this study, but this requires further examination.

The *Listeria *pathogenicity island 1 (LIPI-1) encodes six significant virulence-associated loci: *prfA *(pleiotropic virulence transcriptional regulator), *plcA, plcB *(both encoding phospholipases), *hly *(listeriolysin O), *mpl *(metalloprotease) and *actA *(involved in actin-mediated motility). Previous phylogenetic analysis of this region from food, clinical, animal and environmental isolates indicated that this gene cluster was genetically diverse and clustered based on the three *L. monocytogenes *evolutionary lineages [25,45]. BLASTn comparative analyses of this ~9 kbp region from 08-5578 and 08-5923 identified a very high sequence identity with other lineage II isolates, including 99.5% identity to the EDGe reference genome; ≥ 98% identity to all 23 lineage II isolates characterized by Ward *et al. *[25], and ≥ 98% nucleotide identity to all 21 lineage II isolates characterized by Orsi *et al. *[45]. In comparison, all LIPI-1 regions encoded by evolutionary lineage I and III strains in these previous studies were ≤ 95% identical to our lineage II strains.

PrfA regulates additional virulence determinants encoded elsewhere on the chromosome, such as *bsh *encoding a bile salt hydrolase that promotes survival within the gut [46] and *uhpT *encoding for a hexose phosphate permease for utilization of host carbon sources [47]. These latter two determinants were also encoded within 08-5578 and 08-5923. The presence of these determinants along with LIPI-1 is well conserved across *L. monocytogenes *strains independent of evolutionary lineage, so are unlikely to enhance virulence potential unless the allelic diversity observed between lineages results in a phenotypic change. In general, the contribution of lineage-specific genome differences to virulence and pathogenicity of *L. monocytogenes *are not well understood. For example, recent *in silico *analyses have indicated that while there are lineage II-specific genomic regions not present in lineage I, their role in virulence is not clear [30]. Furthermore, the presence of the *Listeria *pathogenicity island LIPI-3 operon encoding Listeriolysin S has been associated with evolutionary lineage I strains causing foodborne outbreaks [48]. This region was absent in the genomes of both 08-5578 and 08-5923.

### Outbreak isolates harbor a putative mobile genetic island encoding translocation functions

A large 49.8 kbp contiguous region (coordinates 1836435-1886209 of 08-5578; coding sequences LM5578_1850 to LM5578_1903) was present in both sequenced genomes yet absent in all publicly available *Listeria *genome sequences to date, including EGDe (Fig. 2). The bordering coding sequences LM5578_1849 and LM5578_1904 were each 98% identical to contiguous EGDe coding sequences lmo1702 and lmo1703, respectively, implying that the 50 kbp operon-like structured region represents a genomic insertion within the ancestral chromosome of these isolates. Accordingly, putative serine recombinases are encoded in this region (loci LM5578_1855-58) and 16 bp imperfect inverted repeats are present at the borders in the intergenic regions between loci 1849/50 and 1903/04 (Fig. 5B), indicating that this is a horizontally acquired genetic island, hereafter designated *Listeria *genomic island 1 (LGI1).

Coding sequences within LGI1 exhibited sequence homology and were similarly organized as contiguous regions present within several environmental firmicutes, including *Clostridium kluyveri*, *C. bolteae *and *Desulfitobacterium hafniense*. These LGI1-like regions also appeared to be horizontally-acquired, based on skewed G/C content relative to neighboring sequences [49]. The genetic organization and predicted functions of several LGI1 loci resembled the proposed *B. anthracis *pXO1 plasmid-encoded secretion system [50], including several coding sequences that resembled putative type II and type IV secretion systems (T4SS) (Fig. 5B; and Additional file 2). Prototypical members of these systems are involved in pilus biogenesis and translocation of DNA-protein complexes or virulence effectors, respectively. Canonical T4SS genes predicted within LGI1 include *virB4, virD4*, and *virB11*, which encode ATPases involved in substrate recruitment to the transfer complex and substrate translocation, and *virB5 *and *virB6 *subunit genes, which form the core membrane-spanning transfer complex (Fig. 5B; and Additional file 2). Genes that likely contribute to pilus-like surface appendages were also detected (*cpa*, *tad*), and the presence of a *dnaG *gene encoding a putative primase neighboring the *virD4 *coupling protein homolog suggests that this genetic island may be mobilizable (see Additional file 2). Besides pXO1, Type IV secretion-like systems (T4SLS) features have also been described in Gram-positive plasmids such as *B. anthracis *plasmid pXO2 [51,52] and the pheromone-inducible conjugative plasmid pCF10 of *Enterococcus faecalis *[53].

Gram-positive T4SLS also encode a putative peptidoglycan hydrolase and adhesin, both of which are predicted functions in LGI1 (LM5788_1873 and LM5578_1866, respectively). LM5788_1873 encodes a C-terminal NlpC/P60 domain, and is predicted to be involved in assembly of the core substrate transfer complex as a VirB1-like peptidoglycan hydrolase. A P60 domain is also present in the autolytic virulence determinant designated *Listeria *invasion associated protein (Iap) [54]. LM5578_1866 is predicted to encode a Sel1-like repeat (SLR) family protein that contains helical domains mediating protein-protein interactions [55]. Although protein adhesins are often used in Gram-positive bacteria to mediate conjugative attachment to target cells, SLR proteins also are involved for eukaryotic cell entry by *Legionella pneumophilia *[56,57]. Whether this T4SLS is involved in translocation of pathogenicity effector molecule(s) (in addition to probable DNA-protein translocation) is tempting to speculate, but further study is required.

LGI1 also encoded a homolog (LM5578_1862) to the multidrug efflux proton:drug antiporter EmrE implicated in resistance to toxic cationic hydrophobic compounds such as quaternary ammonium compounds and tetracycline [58]. This gene was flanked by a MarR-family transcriptional regulator loci (1425) and a putative DNA-directed RNA polymerase sigma-24 subunit (*rpoE*, 1861) encoding a specialized ECF (extracytoplasmic function) family sigma factor, which is part of the bacterial stress response regulon [59]. A two component signal transduction system (sensor histidine kinase and response regulator; LM5578_1852 and 1851) and restriction modification components (LM5578_1850 and 1853) also were present. Cumulatively, LGI1 is unique compared to all currently sequenced *L. monocytogenes *but the contribution to pathogenicity or environmental persistence is unconfirmed.

## Conclusions

High-throughput DNA sequencing rapidly provided the complete genetic content of two *L. monocytogenes *outbreak isolates. Within three days of project commencement, draft genome sequences were available that were suitable to begin comparative analyses such as genome alignments, preliminary annotation of coding sequences, and identification of traits associated with macro-diversity and micro-diversity. This allowed us to determine evolutionary lineages and unequivocally define the full breadth of genetic variation between two subtype variants identified by the internationally PulseNet standardized PFGE typing method. Whole genome sequencing therefore enabled robust real-time characterization of virulence determinants and genetic diversity (prophage and plasmid elements; SNP and indel mutations) within a natural *L. monocytogenes *population. These novel markers were then applied for a rapid assessment of the genetic relatedness of additional clinical, food, and environmental isolates recovered during the outbreak. The distribution of the SNP, indel and prophage traits indicated that three distinct but highly related strains were likely involved. Further characterization of the SNPs indicated that clinical isolate 08-5923 was likely subjected to positive selective pressures resulting in a higher frequency of non-synonymous mutation than would normally be expected for *L. monocytogenes*. Selective pressure for adaptive change could have resulted from host specific conditions during infection or adverse food storage conditions, but with only a single available isolate representative of this SNP genotype it is not possible to identify the micro-diversification timeline.

Previous comparative genomic studies of outbreak-associated *L. monocytogenes *have found several strain and serotype-specific coding sequences between serotype 4b and 1/2a strains, but amongst these there were few relevant genetic traits suggestive of virulence potential [21,30]. One notable exception was variation in the complement of internalin-like coding determinants [21], and consistent with this finding, a large composition of internalin-like determinants was observed in the current study. In addition, there were two large-scale genetic insertions unique to our serotype 1/2a strains. Prophage ϕLMC1 was composed of genes related to previously characterized phage determinants, but on the whole, represented a novel *Listeria *phage. A ~50 kbp genetic island (LGI1) unique amongst all other currently sequenced *L. monocytogenes *isolates was also present in 08-5578, encoding putative translocation, resistance, and regulatory determinants.

While *L. monocytogenes *serotype 1/2a can be frequently isolated from food processing environments, the majority of invasive listeriosis outbreaks to date have resulted from lineage I serotype 4b strains [15]. Lineage II serotype 1/2a strains have more frequently been associated with outbreaks of listerial gastroenteritis [15]. Our current study demonstrates that lineage II strains can also cause large outbreaks of severe invasive disease and is consistent with a global trend towards serotype 1/2a predominance. Whole genome sequencing allowed us to detect within these outbreak isolates a repertoire of genetic determinants involved in diversification and microevolution. These features may have a role in virulence and pathogenicity, as well as survival within food processing environments and in foods. Genomic studies can therefore facilitate a greater understanding of the lineage-specific and strain-specific features of *L. monocytogenes *and how they contribute to this pathogen's ecology and virulence. This knowledge may ultimately lead to the development of methods to better assess the risks posed by individual *L. monocytogenes *strains.

This study also provides a proof-of-concept that the latest generation DNA sequencing platforms have a place in real-time public health responses to bacterial pathogens. Whole genome sequencing may not be ready to be applied routinely as a subtyping method, but public health laboratories need to prepare for this inevitability and assess how to properly analyze and interpret whole genome sequences in the context of molecular epidemiology. Most of the current subtyping methods such as PFGE and MLST capture only a small proportion of the true genetic content, so it is still difficult to interpret robust data sets such as whole genomes when all previous characterizations have been comparatively limited in detail. It is foreseeable that the burgeoning capacity for whole genome sequencing will soon provide a cost effective alternative to the current subtyping methods after a reconciliation has been made between the true nature of subtypes, the diversity revealed by classical methods and the micro- and macro-diversity readily identified by next-generation sequencing technologies. As more genomes from clinical isolates are completed and compared (as in this study), it will become increasingly feasible to apply genome sequencing for responses to bacterial outbreaks.

## Methods

### Bacterial isolates

Clinical, food and environmental isolates (Table 1) were collected by the Ontario Central Public Health Laboratory and the Canadian Food Inspection Agency during an outbreak investigation of *L. monocytogenes *and transferred to the National Microbiology Laboratory (NML) for additional subtyping. The clinical isolates examined in this study were collected from individual outbreak-associated cases.

### Serotyping and Pulsed-field Gel Electrophoresis

Serotyping was performed by slide agglutination with antisera prepared at the NML according to Seeliger and Höhne [60]. Molecular serotyping was completed using a multiplex PCR scheme [61]. Pulsed-field gel electrophoresis (PFGE) was performed according to the PulseNet standardized protocol using restriction enzymes *Asc*I and *Apa*I. PFGE patterns were designated using BioNumerics after comparison to the PulseNet Canada database.

### Genome sequencing and bioinformatics

Genome sequencing was performed on the Roche GS FLX™ standard platform, as per the manufacturer's recommendations. The relative genome coverage achieved was approximately 40× for 08-5578 (Q40+ bases = 99.88%) and 36× for 08-5923 (Q40+ bases = 99.91%). Within the 08-5578 project the coverage of pLM5578 was 68×, indicating that the plasmid copy number was between 1-2 copies per cell. *De novo *sequence assembly was completed using Roche's Newbler assembler and the Staden software package [62] after a fosmid mate-pair end library was constructed. Gap closure was completed by Sanger-based sequencing of select regions to bridge contigs. Presumptive insertion/deletions (indels) associated with homopolymeric regions were verified or corrected after Sanger-based sequencing of directed PCR amplicon templates generated from the corresponding regions in both 08-5578 and 08-5923 by proof-reading Taq polymerase. Sequences were deposited into GenBank under the accession numbers CP001602 (08-5578), CP001603 (pLM5578) and CP001604 (08-5923).

Pyrosequencing was completed within three days to provide greater than 99% of the genome coverage. This was sufficient for preliminary analysis such as identification of plasmid and phage determinants and candidate SNP's. Closed circular chromosomal sequences were obtained within an additional 5 weeks after fosmid libraries were constructed, contigs ordered and all gaps closed. Three additional weeks were required for resequencing of SNP and homopolymeric regions to confirm polymorphisms and indels between the two sequenced isolates. The costs associated with this project (in US dollars) were $5000 for the GS-FLX run covering both isolates, $500/isolate for the fosmid libraries and related consumables, and $2000/isolate for primer synthesis and Sanger-based sequencing for gap closure and resequencing.

Annotation of coding sequences for isolate 08-5578 was completed using GenDB v2.2 [63], BASys [64], Glimmer3 [65] and by comparison to RefSeq annotations completed for *L. monocytogenes *strain EGDe [22]. Sequences encoding rRNA and tRNA were identified by RNAmmer version 1.2 [66] and tRNAscan-SE version 1.23 [67], respectively. Artemis was used for manual annotation and additional manipulations [68]. Multiple sequence alignments of whole genomes and select loci were generated using MAUVE [69] and ClustalW [70], respectively, and maximum likelihood phylogenetic trees were generated using the Phylogeny Inference Package (Phylip version 3.68). Distant homologies between hypothetical coding sequences and entries at the PDB, COG, PFAM and SCOP databases were detected using the FFAS03 server [71]. Single nucleotide polymorphisms (SNPs) were identified between the sequenced isolates using NUCMER and dot plots were generated using MUMmerplot [72]. The circular representation of the genome of 08-5578 was created using DNAplotter [73] and feature tables generated using Artemis.

Reference *Listeria *genome sequences were obtained from GenBank for isolates EGDe (accession number NC_003210), F2365 (NC_002973), HCC23 (NC_011660), Clip11262 (NC_003212; *L. innocua*), SLCC5334 (NC_008555; *L. welshimeri*) and *L. monocytogenes *phage B025 (NC_009812). Accession numbers for plasmid DNA sequences of pLI100 and pXO2 were NC_003383 and NC_001496, respectively. In addition, the DNA sequences of *L. monocytogenes *strains F6854 and H7858 [21] and plasmid pLM80 from strain H7858 were obtained from the J. Craig Venter Institute Comprehensive Microbial Resource http://cmr.jcvi.org/cgi-bin/CMR/CmrHomePage.cgi. A cross match table (Additional file 5) of the coding sequences between all of these *L. monocytogenes *genomes was generated using the publically available annotations as of September 18^th^, 2009 with a reciprocal BLASTp alignment threshold of 80% identity and 80% hsp. Draft genomes sequences at the Broad Institute were also screened for select regions using BLAST available at their database http://www.broad.mit.edu/annotation/genome/listeria_group/MultiHome.html. MLST loci data were downloaded from the Institute Pasteur *Listeria monocytogenes *MLST Database http://www.pasteur.fr/recherche/genopole/PF8/mlst/Lmono.html.

### PCR screening and SNP confirmation

All polymerase chain reactions were performed with Invitrogen HiFi Platinum proof-reading Taq polymerase following the manufacturer's directions, and with 1 uM of each oligonucleotide (see Additional file 6) using the following thermocycling conditions: 94°C for 5 min.; 35 cycles of 94°C for 30 sec., 50°C for 30 sec. and 68°C for 45 sec.; followed by 68°C for 5 min. Amplicons were visualized after electrophoresis in 1% agarose-TBE gels. Confirmation of SNP sites was achieved by Sanger-based sequencing of targeted amplicons using the same oligonucleotides as used for PCR amplification.

## Authors' contributions

Conceived and designed the experiments: MWG ST MG KT-Y OL GVD CN. Performed the experiments: ST KT-Y OL CN. Analyzed the data: MWG MG GVD ST HK KT-Y OL VA BA CN. Contributed reagents/materials/analysis tools: VA BA GVD HK. Wrote the paper: MWG MG GVD CN. Edited the manuscript: MWG ST MG GVD HK KT-Y OL VA BA CN. All authors read and approved the final manuscript.

## Supplementary Material

Additional file 1 Identification of bacteriophage-related sequences in the putative prophage ϕLMC1. Click here for file

Additional file 2 **Coding sequences on plasmid pLM5578 and the *Listeria *genetic island (LGI1) predicted to encode translocation functions**. FFAS03 scores above -9.0 are not significant, but three predictions near this threshold were included. Click here for file

Additional file 3 **Dot plot comparison of collinearity of *Listeria monocytogenes *genome 08-5578 relative to EGDe**. Dots or lines represent segments of conservation between the two sequences. Blue lines represent collinear sequence similarities between the reverse complement of 08-5578 and EGDe and represent a symmetrical inversion around the origin of replication. Outlier dots indicate duplicated regions. A break in the main diagonal of a linear segment supports an insertion or deletion in either one of the sequences, and the associated features are identified with arrows ('LGI1', *Listeria *genomic island 1). Click here for file

Additional file 4 **Internalin and Internalin-like leucine rich coding sequences present in isolate 08-5578**. The conserved LPxTG cell wall anchor domains were identified using HMMER TIGR01167. Click here for file

Additional file 5 **Cross-match table of all protein-encoding loci encoded by two serotype 1/2a outbreak-associated *Listeria monocytogenes *(08-5578 and 08-5923) versus other publically available, annotated *L. monocytogenes *genomes**. Description and references are included in the Methods section. Click here for file

Additional file 6 **Oligonucleotides used in this study**. All coordinates are in reference to the sequence of isolate 08-5578. Click here for file
