# Supplementary figures and images for: High-throughput genome sequencing of two Listeria monocytogenes clinical isolates during a large foodborne outbreak
Source: BMC Genomics. 2010 Feb 18;11:120. doi: 10.1186/1471-2164-11-120 (PMC2834635; doi:10.1186/1471-2164-11-120)

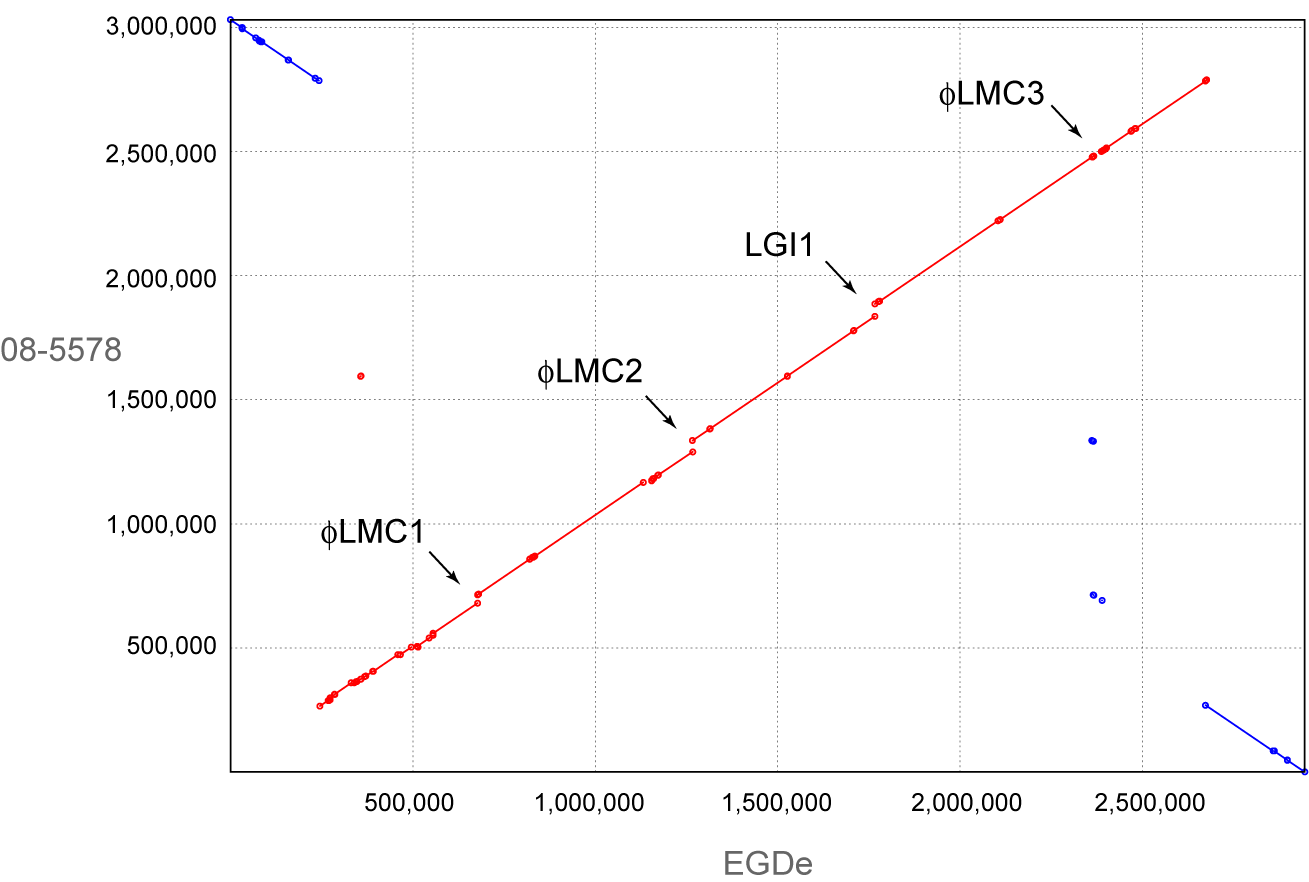

Supplement: Additional file 3 — Dot plot comparison of collinearity of Listeria monocytogenes genome 08-5578 relative to EGDe. Dots or lines represent segments of conservation between the two sequences. Blue lines represent collinear sequence similarities between the reverse complement of 08-5578 and EGDe and represent a symmetrical inversion around the origin of replication. Outlier dots indicate duplicated regions. A break in the main diagonal of a linear segment supports an insertion or deletion in either one of the sequences, and the associated features are identified with arrows ('LGI1', Listeria genomic island 1). [file 1471-2164-11-120-S3.TIFF]
