# Supplementary material for: High-throughput genome sequencing of two Listeria monocytogenes clinical isolates during a large foodborne outbreak
Source: BMC Genomics. 2010 Feb 18;11:120. doi: 10.1186/1471-2164-11-120 (PMC2834635; doi:10.1186/1471-2164-11-120)
Supplement: Additional file 6 — Oligonucleotides used in this study. All coordinates are in reference to the sequence of isolate 08-5578. [file 1471-2164-11-120-S6.DOC]

**Additional file 6.** Oligonucleotides used in this study. All coordinates are in reference to the sequence of isolate 08-5578.

| **Oligonucleotide** | **Target** | **Sequence (5' to 3')** |
| --- | --- | --- |
|  |  |  |
| GIL362 | pLM5578; *virD4* | GATCCGAAAGGGGAATTAGC |
| GIL363 |  | GCAATTGGCTTTTTCTCTGC |
|  |  |  |
| GIL364 | pLM5578; *fic* | AGTGCGATTGAAGGGAACAC |
| GIL365 |  | AGCCCGAAACATCTTGATTG |
|  |  |  |
| GIL366 | *gltX* | ACTTCCGCAGCTTCAAGAAC |
| GIL367 | (LM5578_0279) | ATCGCTATGCTTGGTTGGTC |
|  |  |  |
| GIL368 | Phage terminase | GTTCATTATTCCGCTCTCAAGG |
| GIL369 | (LM5578_0693) | TTTTATCCAGGTCGTTTCGTCT |
|  |  |  |
| GIL370 | Phage tail | GAAACTTCTTGCAGCGGAAC |
| GIL371 | (LM5578_0704) | GCGTTTGAATTCCCAGTTGT |
|  |  |  |
| Lm65f | *L. monocytogenes* | CGTGCTGAAACTGACCAAGA |
| Lm65r | SNP Position 213957 | CATTGAAGCAGCCACTTTGA |
|  |  |  |
| Lm103f | *L. monocytogenes* | CCGTTTGTCTGACGCACTAA |
| Lm103r | SNP Position 172841 | AACTGCGGATAAAGCGAGAA |
|  |  |  |
| Lm164f | *L. monocytogenes* | CTACGTACTGCTCGCTGCTG |
| Lm164r | SNP Position 113283 | GCTAACCATCCACCGAAGAA |
|  |  |  |
| Lm226f | *L. monocytogenes* | AAGGGGTGGAAAAGTTCCTG |
| Lm226r | SNP Position 47737 | CATTGAATCCGCCACTTCTT |
|  |  |  |
| Lm364f | *L. monocytogenes* | CCCTTACCGCGCAATATTTA |
| Lm364r | SNP Position 2929661 | TCATCAAGTGCATTCCCAAA |
|  |  |  |
| LmI1f | *L. monocytogenes* | GCCAGACCATGCTTTTGTTT |
| LmI1r | SNP Position 2691224 | CACGGTTAAAACCAGCCAAT |
|  |  |  |
| Lm678f | *L. monocytogenes* | AATGCTGCCTTTCTGCTCAT |
| Lm678r | SNP Position 2603137 | TCCTTCCAGACCAACCAATC |
|  |  |  |
| Lm890f | *L. monocytogenes* | CGGGTTCATAATGGTTTTCG |
| Lm890r | SNP Position 2404315 | AAACCGTCCATCCCAACATA |
|  |  |  |
| LmI2f | *L. monocytogenes* | TGCTGAACTGGCACGATTAG |
| LmI2r | SNP Position 2261927 | GCGATGTGAAAGCAACTGAA |
|  |  |  |
| Lm1134f | *L. monocytogenes* | AAGAAGCCGAGCGACAATAA |
| Lm1134r | SNP Position 2146277 | CTGGTGGTGTGAACGAATTG |
|  |  |  |
| Lm1156f | *L. monocytogenes* | CACCACATCATCATGCACAA |
| Lm1156r | SNP Position 2124375 | GTGTTGCGTGAAATGTACCG |
|  |  |  |
| Lm1163f | *L. monocytogenes* | GCCTGGCACTGTCATTTCTT |
| Lm1163r | SNP Position 2117257 | TATTGTGCATGGCACTGGTT |
|  |  |  |
| Lm1358f | *L. monocytogenes* | GCGTGGCTATTCCATTTGTT |
| Lm1358r | SNP Position 1910870 | TATGCAACGGATGGTTTTGA |
|  |  |  |
| Lm1365f | *L. monocytogenes* | TGGGTTGGTAACTGGACCAT |
| Lm1365r | SNP Position 1901622 | CCTTACCATCCCTTGCAGAA |
|  |  |  |
| Lm1474f | *L. monocytogenes* | TAAAAATGGCCGAAAATTGC |
| Lm1474r | SNP Position 1787404 | GCATCCCAGCATTAGGTGAT |
|  |  |  |
| Lm1587f | *L. monocytogenes* | ACCCATATTTCCTGCGACTG |
| Lm1587r | SNP Position 1660409 | CGCTGATCCAGCTGTTGTTA |
|  |  |  |
| Lm1776f | *L. monocytogenes* | GGCGCAGGAAGTAGTACAGC |
| Lm1776r | SNP Position 1462121 | GTAAACCGCGAGCTTGGTAG |
|  |  |  |
| Lm1927f | *L. monocytogenes* | GCAGATGAAGTCGCTGACAA |
| Lm1927r | SNP Position 1319530 | CACCTGGTGAAGGACCACTT |
|  |  |  |
| Lm2170f | *L. monocytogenes* | AAAAGGCTGGAATCCAAGGT |
| Lm2170r | SNP Position 1096500 | GCGCAAGGCATAATACCAAT |
|  |  |  |
| Lm2190f | *L. monocytogenes* | AGCCGGGAAACCAACTTACT |
| Lm2190r | SNP Position 1076779 | TCATCGTACCGTCACCGATA |
|  |  |  |
| Lm2339f | *L. monocytogenes* | ATACCAGTGCGCGAATTACC |
| Lm2339r | SNP Position 940888 | CTGCCTGCACTGCATCTTTA |
|  |  |  |
| LmI4f | *L. monocytogenes* | AGTATCGCGACGATTTGCTT |
| LmI4r | SNP Position 850721 | CATCCCTTTCGTTACGCACT |
|  |  |  |
| Lm2435f | *L. monocytogenes* | TGGAATGATCGCACTTGAAA |
| Lm2435r | SNP Position 833717 | AATTCAACCGTTTCCATCCA |
|  |  |  |
| Lm2666f | *L. monocytogenes* | ACTTGGGACGAAGCATTCAC |
| Lm2666r | SNP Position 630182 | CTTTAAAATTCGCCGTTCCA |
|  |  |  |
| Lm2717f | *L. monocytogenes* | CTCGGGAAACAGTTGCTGAT |
| Lm2717r | SNP Position 577443 | TCAATGTTCGCTGCTTTGAC |
|  |  |  |
| Lm2737f | *L. monocytogenes* | GCGACGGAAATGTTTTAGGA |
| Lm2737r | SNP Position 552482 | AGCTGGATCCTTTGCGTCTA |
|  |  |  |
| Lm2984x1f | *L. monocytogenes* | GTGAATATTCGGCGCAAGTT |
| Lm2984x1r | SNP Position 291653 | GCTTGAATGGAGGTTGGTGT |
|  |  |  |
| Lm2984x2f | *L. monocytogenes* | ATTCGGCGCAAGTTCTATTG |
| Lm2984x2r | SNP Position 291652 | CGCAGCTTCTGGATCTTTCT |
